# Supplementary material for: A Nutrigenetic Strategy for Reducing Blood Lipids and Low-Grade Inflammation in Adults with Obesity and Overweight
Source: Nutrients. 2023 Oct 10;15(20):4324. doi: 10.3390/nu15204324 (PMC10609523; doi:10.3390/nu15204324)
Supplement: Supplementary file 1 [file nutrients-15-04324-s001.zip › nutrients-2661494-supplementary.pdf]

nutrients

MDPI

Supplementary Materials

Table S1. General Information about genotyping assay.

Gene	Name	SNP ID	Location	Catalog number	Contetext Sequence [VIC/FAM]
FABP2	Fatty acid binding protein 2	rs1799883	Chr.4:119320747	4351379 C____761961_10	AACAACTTCAATGTTTCGAAAAG**[C/T]**GCTTGATTCTTTGACTGTG AATTTA
PPARG	Peroxisome proliferator-activated receptor gamma	rs1801282	Chr.3:12351626	4362691 C___1129864_10	AACTCTGGGAGATTCTCCTATTGAC**[C/G]**CAGAAAGCGATTCCTTC ACTGATAC
LPL	Lipoprotein Lipase	rs13702	Chr.8:19966981	4351379 C___9639448_10	GGAACTCTGGCTCCGAAAAACTTTG**[C/T]**TATATATATCAAGGATG TTCTGGCT
APOA5	Apolipoprotein A5	rs662799	Chr.11:116792991	4351379 C___2310403_10	GAGCCCCAGGAACTGGAGCGAAAGT**[A/G]**AGATTTGCCCCATGAG GAAAAGCTG
APOA1	Apolipoprotein A1	rs670	Chr.11:116837697	4351379 C___2679585_20	GCTGGGAGGCTGATAAGCCCAGCCC**[C/T]**GGCCCTGTT-GCTGCTCACTGGTCCT
APOC3	Apolipoprotein C3	rs5128	Chr.11:116832924	4351379 C___8907537_1_	AAGTCCACCTGCCTATCCATCCTGC**[C/G]**AGCTCCTTGGGTCCTG-CAATCTCCA
APOE	Apolipoprotein E	rs7412	Chr.19:44908822	4351379 C____904973_10	CCGCGATGCCGATGACCTGCAGAAG**[C/T]**GCCTGGCAGTGTAC-CAGGCCGGGGC
		rs429358	Chr.19:44908684	4351379 C___3084793_20	GCTGGGCGCGGACATGGAGGACGTG**[C/T]**GCGGCCGCCTGGTGCA GTACCGCGG
ABCA1	*ATP binding cassette transporter 1*	rs9282541	Chr.9:104858554	4351379 C__11720861_10	GGCTTCAGGATGTCCATGTTGGAAC**[A/G]**AAGTACTCGCTCTGCTG CAGCCAGT
CETP	Cholesteryl ester transfer protein	rs708272	Chr.16:56962376	4351379 C___9615318_10	CTGAGACCCAGAATCACTGGGGTTC**[A/G]**AGTTAGGGTTCAGATCT GAGCCAGG
LIPC	Lipase C. Hepatic	rs1800588	Chr.15:58431476	4351379 C___8757333_30	GCTTCTTCGTCAGCTCCTTTTGACA**[C/T]**GGGGGTGAAGGGTTTTCT GCACCAC

SNP Genotyping/ TaqMan Assays ThermoFisher Scientific: https://www.thermofisher.com.

Table S2. Genotypic frequencies (GF) of the study population vs. reported in database.

Gene	SNP	Alleles	MAF[1]	MAF Reported	HWE
LIPC	rs1800588	C/T	0.472 (T)	0.461(T)	0.915
LPL	rs13702	T/C	0.260 (C)	0.278 (C)	0.968
FABP2	rs1799883	G/A	0.252 (A)	0.260 (A)	0.906
ABCA1	rs9282541	C/T	0.068 (T)	0.120 (T)	0.838
CETP	rs708272	G/A	0.412 (A)	0.467 (A)	0.997
PPARG	rs1801282	C/G	0.132 (G)	0.113(G)	0.811
APOA5	rs662799	T/C	0.156 (C)	0.160 (C)	1.000
APOC3	rs5128	C/G	0.184 (G)	0.172 (G)	0.764
APOA1	rs670	G/A	0.272 (A)	0.300 (A)	0.730
APOE	rs429358	T/C	0.124 (C)	0.079 (C)	0.674
	rs7412	C/T	0.040 (T)	0.018 (T)	0.984

MAF[1]: Minor Allele Frequency regarding this study population; MAF Reported from 1000 Genomes and the International HapMap project databases. HWE: Hardy-Weinberg equilibrium; HWE is expressed as p value.

Table S3. Genotype codifications of single specific SNPs statistically associated with TGs and VLDL-c decrease by diets.

STANDARD DIET									NUTRIGENETIC DIET								
***LPL* rs13702**									***LIPC* rs1800588**								
	**ΔTGs**				**ΔVLDL**					**ΔTGs**				**ΔVLDL**			
	n	Mean	Std error	*p* value	n	Mean	Std error	*p* value		n	Mean	Std error	*p* value	n	Mean	Std error	*p* value
Risk (TC + CC)	2	90.50	34.50	0.08	2	18.00	7.00	0.08	Risk (CT+ TT)	12	73.42	26.72	**0.006**	12	14.75	5.32	**0.006**
Non risk (TT)	34	9.26	10.80		34	1.91	2.15		Non risk (CC)	30	16.40	6.70		30	3.33	1.35	
***APOA1* rs670**																	
	**ΔTGs**				**ΔVLDL**												
	n	Mean	Std error	*p* value	n	Mean	Std error	*p* value									
Risk (GA+ AA)	4	-44.0	30.46	0.56	4	-8.75	6.06	0.55									
Non risk (GG)	32	21.0	10.98		32	4.25	2.18										

Data presented as mean ± Standard error. Bold numbers indicate $p < 0.05$. ΔTGs: delta of triglycerides; ΔVLDL: delta of very-low density lipoprotein cholesterol.
